# Deep learning model for the early prediction of pathologic response following neoadjuvant chemotherapy in breast cancer patients using dynamic contrast-enhanced MRI

**DOI:** 10.3389/fonc.2025.1491843

**Published:** 2025-02-25

**Authors:** Meng Lv, BinXin Zhao, Yan Mao, Yongmei Wang, Xiaohui Su, Zaixian Zhang, Jie Wu, Xueqiang Gao, Qi Wang

**Affiliations:** ^1^ Breast Disease Diagnosis and Treatment Center, The Affiliated Hospital of Qingdao University, Qingdao, Shandong, China; ^2^ Department of Radiation Oncology, The Affiliated Hospital of Qingdao University, Qingdao, Shandong, China; ^3^ Department of Radiology, The Affiliated Hospital of Qingdao University, Qingdao, Shandong, China; ^4^ Department of Pathology, The Affiliated Hospital of Qingdao University, Qingdao, Shandong, China

**Keywords:** breast cancer, neoadjuvant chemotherapy, Miller-Payne grading criteria, dynamic contrast enhancement MRI, deep learning model

## Abstract

**Purpose:**

This study aims to investigate the diagnostic accuracy of various deep learning methods on DCE-MRI, in order to provide a simple and accessible tool for predicting pathologic response of NAC in breast cancer patients.

**Methods:**

In this study, we enrolled 313 breast cancer patients who had complete DCE-MRI data and underwent NAC followed by breast surgery. According to Miller-Payne criteria, the efficacy of NAC was categorized into two groups: the patients achieved grade 1-3 of Miller-Payne criteria were classified as the non-responders, while patients achieved grade 4-5 of Miller-Payne criteria were classified as responders. Multiple deep learning frameworks, including ViT, VGG16, ShuffleNet_v2, ResNet18, MobileNet_v2, MnasNet-0.5, GoogleNet, DenseNet121, and AlexNet, were used for transfer learning of the classification model. The deep learning features were obtained from the final fully connected layer of the deep learning models, with 256 features extracted based on DCE-MRI data for each patient of each deep learning model. Various machine-learning techniques, including support vector machine (SVM), K-nearest neighbor (KNN), RandomForest, ExtraTrees, XGBoost, LightGBM, and multiple-layer perceptron (MLP), were employed to construct classification models.

**Results:**

We utilized various deep learning models to extract features and subsequently constructed machine learning models. Based on the performance of different machine learning models’ AUC values, we selected the classifiers with the best performance. ResNet18 exhibited superior performance, with an AUC of 0.87 (95% CI: 0.82 - 0.91) and 0.87 (95% CI: 0.78 - 0.96) in the train and test cohorts, respectively.

**Conclusions:**

Using pre-treatment DCE-MRI images, our study trained multiple deep models and developed the best-performing DLR model for predicting pathologic response of NAC in breast cancer patients. This prognostic tool provides a dependable and impartial basis for effectively identifying breast cancer patients who are most likely to benefit from NAC before its initiation. At the same time, it can also identify those patients who are insensitive to NAC, allowing them to proceed directly to surgical treatment and prevent the risk of losing the opportunity for surgery due to disease progression after NAC.

## Introduction

Breast cancer has become the most common prevalent malignancy worldwide and the first leading cause of cancer death in women (1). Neoadjuvant chemotherapy (NAC) is recommended as the standard treatment for both locally advanced and early invasive breast cancer patients with an intent to perform breast-conserving surgeries (2). The evaluation of NAC also provided prognosis prediction and *in vivo* drug susceptibility test. Research has indicated that a significant proportion of patients may experience beneficial effects from NAC, potentially achieving a complete pathologic response (pCR). Nevertheless, a subset of 10-35% of breast cancer cases have been identified as unresponsive to NAC, with approximately 5% of patients exhibiting tumor growth following treatment (3). In such cases, NAC has been shown to be ineffective and may even delay surgical intervention. Therefore, early prediction of response to NAC is critical for optimizing and adjusting therapeutic strategies, which may mitigate toxicity without impacting efficacy. The Miller-Payne grading criteria serves as a suitable pathological assessment method, utilizing tumor cell density and morphology to classify residual tumors as Grade 1-5. Grade 4-5 tumors are characterized by no evidence of residual tumor or microscopic foci of invasive carcinoma, and are indicative of chemotherapy-sensitive breast cancers with a optimistic long-term prognosis.

Dynamic contrast enhancement MRI (DCE-MRI) is the most common and effective imaging test for clinical breast MRI examinations. It has shown superiority of identifying small breast cancer lesions, and evaluating blood perfusion and distribution of tumor vessels. Therefore, DCE-MRI is recommended to evaluate the efficacy of NAC in breast cancer patients following an early treatment period (4). Previous studies investigated the role of quantitative DCE-MRI parameters in the therapeutic evaluation of NAC. A retrospective study enrolled 37 breast cancer patients and found changes in DCE-MRI kinetic parameters were correlated with pathologic response after NAC (5). Li and colleagues (6) discovered the signal enhancement ratio washout volume and K_ep_ might prognosticate pathologic response in breast cancer patients. The diagnostic efficacy of quantitative parameters ranged from 0.73 to 0.78. The efficacy of DCE-MRI in prediction of pathologic response was limited and relied on dynamic changes of radiologic parameters. Importantly, the reliable volumetric and kinetic parameters in the prediction of therapeutic efficacy cannot require prior to NAC treatment (7).

Radiomics, an emerging field in cancer treatment, involves the automated analysis of quantitative data extracted from medical images to correlate with malignant biological properties, therapeutic efficacy, and clinical prognosis. This approach offers the potential for individualized precision therapy in a non-invasive manner, allowing for the characterization of tumor properties solely through imaging data rather than invasive sampling procedures. Advancements in deep learning radiomics (DLR) and data processing tools have facilitated the interpretation and utilization of data in clinical settings. Unlike traditional radiomics methods, deep learning-based radiomics techniques exploit the inherent non-linearity of deep neural networks to extract relevant features automatically without manual feature extraction. On the other hand, deep learning has the capability to leverage comprehensive feature data, particularly with respect to the spatial arrangement of pixels, in order to extract information pertaining to the textures and shapes. Consequently, even when employing basic digital images, deep learning is anticipated to excel in the precise and detailed identification. Verma (8) et al. investigated a multimodal spatiotemporal DLR to predict pCR of NAC among breast cancer patients. The AUC of 3D-VGGNet and 3D-ResNet signatures were 0.68, and 0.50, respectively. Due to the limited prognostic efficacy, many studies focused on the fusion of different DLR models with multimodal images, which complicated the development of predictive signature. In a retrospective study, 536 breast cancer patients were enrolled to provide a DLR signature for predicting pCR to NAC (9). The fusion of different DLR signatures with multiple MR images yielded an AUC of 0.745. Although DLR has been proposed for predicting pathologic response following NAC, these studies have been hindered by small sample sizes and limited predictive accuracy. Meanwhile, most of these studies focused on the prediction of pCR, instead of pathologic response, following NAC in breast cancer patients. Hence, this study aims to investigate the diagnostic accuracy of various deep learning methods on DCE-MRI, in order to provide a simple and accessible tool for predicting pathologic response of NAC in breast cancer patients.

## Materials and methods

### Patients

A total of 313 newly diagnosed breast cancer patients treated at the Affiliated Hospital of Qingdao University between 2016 and 2020 were included in this retrospective study, for which informed consent was waived. The study was approved by the ethics committee of the Affiliated Hospital of Qingdao University and adhered to the principles outlined in the Declaration of Helsinki. The study established specific inclusion criteria, including (1) primary invasive breast cancer confirmed by histology; (2) complete medical records; (3) qualified dynamic contrast-enhanced magnetic resonance imaging (DCE-MRI) images before neoadjuvant chemotherapy (NAC); (4) receipt of preoperative systemic chemotherapy; (5) adherence to NAC protocols based on either the National Comprehensive Cancer Network or Chinese Society of Clinical Oncology guidelines; (6) confirmation of surgical outcomes through pathologic examination of Miller-Payne grading criteria. Concurrently, the exclusion criteria included (1) advanced cancer patients with distant metastases; (2) a prior history of other malignancy, incomplete neoadjuvant chemotherapy (NAC) treatment prior to surgery; (3) incomplete essential clinical data (molecular subtype).

### Pathological evaluation

The patients who underwent surgery following NAC were assessed using the Miller-Payne criteria. The efficacy of NAC was categorized into five levels: G1 denoting some changes in cancer cells without a decrease in total numbers, G2 indicating a reduction rate of <30% with high total numbers, G3 representing a moderate decrease of ≥30% but <90% in cancer cells, G4 showing a significant reduction of ≥90% with only scattered cell clusters remaining, and G5 indicating the absence of cancer cells at the original tumor site. The patients achieved G1, G2, and G3 were classified as the non-responders, while patients achieved G4 and G5 were classified as responders.

### Magnetic resonance acquisition protocol

Pre-treatment dynamic contrast-enhanced magnetic resonance imaging (DCE-MRI) was carried out for each patient prior to biopsy, within a timeframe of 1-2 weeks before NAC. The MRI scan was performed using a 3.0 T scanner equipped with either an 8-channel or 16-channel breast coil (Signa HDxt, GE Healthcare), with patients positioned in a prone manner. The DCE-MRI protocol included one pre-contrast and eight post-contrast T1-weighted images with fat saturation. Following the intravenous administration of gadolinium-DTPA contrast agent (0.2ml/kg), a subsequent flush of 20 ml of saline solution was administered at a flow rate of approximately 2 ml/s. The initial post-contrast images were acquired 60 seconds after the start of the gadolinium-DTPA injection, followed by seven additional scans. The configurations used to obtain MR images were described previously (10).

### Tumor segmentation and deep learning features extraction

The identification and delineation of regions of interest (ROI) were conducted manually on individual slices of DCE-MRI, focusing on the peak enhanced phase determined by the time-intensity curve, utilizing the itk-SNAP software (www.itksnap.org). This task was executed by two radiologists possessing five years of experience each. In the peak enhanced phase of the time-intensity curve, the breast carcinoma exhibited significant enhancement, whereas the surrounding stroma displayed slight enhancement. Subsequent to the completion of tumor masking contouring by the junior radiologist, the senior radiologist boasting 10 years of experience reviewed the ROI for accuracy and implemented any necessary modifications.

Multiple deep learning frameworks, including Vision Transformer (ViT), VGG16, ShuffleNet_v2, ResNet18, MobileNet_v2, MnasNet-0.5, GoogleNet, DenseNet121, and AlexNet, were used for transfer learning of the classification model. In deep learning analysis, a ROI images measuring 448 × 448 pixels was utilized to crop the largest cross-section of breast tumor as input. In order to standardize image signal intensity across patients, image intensity was normalized to a consistent range of 0–1000. The detailed description of the model architectures used in our study was shown in **Supplementary Table S1**. The deep learning process involved the development of independent inputs for each image. Following the completion of training for the deep learning model, features were downscaled from the final fully connected layer to 256 and use them as input for the machine learning model. In the test cohort, ROI images were inputted into the trained deep learning model. The deep learning features from the fully connected layer were also extracted for further analysis.

### Deep learning models construction and validation

The dataset was randomly partitioned into train and test cohorts at an 8:2 ratio. The train cohort was employed for the development of deep learning models utilizing the extracted deep learning features. The radiomic features underwent an initial screening using the Mann-Whitney U test with a significance level set at P < 0.05. Following this, the Pearson correlation coefficient was utilized to assess the correlation between each pair of radiomic features, with features exhibiting a correlation coefficient |r| greater than 0.9 being removed. Feature selection in the train cohort was conducted using the least absolute shrinkage and selection operator (LASSO) method. The settings for the Lasso model are as follows: alpha = 1, and the maximum number of iterations for the optimization algorithm is set to 1000. Various machine-learning techniques, including support vector machine (SVM), K-nearest neighbor (KNN), RandomForest, ExtraTrees, XGBoost, LightGBM, and multiple-layer perceptron (MLP), were employed to construct classification models. A 5-fold cross-validation was performed using the StratifiedKFold function from scikit-learn, which divided the train cohort into five non-overlapping subsets. In each iteration, one partition was used as the test set, while the remaining partitions served as the train set. This approach ensures that each class is represented proportionally across both the training and testing folds, helping to determine the optimal model hyperparameters. The performance of these models was assessed through ROC analysis, as well as the calculation of sensitivity, specificity, positive predictive value(PPV) and negative predictive value(NPV). A calibration curve was utilized to plot prediction probabilities against measured rates. The evaluation of model adequacy was carried out using the Hosmer-Lemeshow test.

### Statistical analyses

Statistical analyses were performed using R Studio (version:2023.12.1) and Python 3.12.2, with the Fisher’s test, χ^2^ test, or Mann-Whitney U test utilized to assess the association between the effectiveness of NAC and clinical variables. “pROC”, “rms”, “rmda”, and “generalhoslem” were used to generate the ROC curve, calibration curve, and Hosmer-Lemeshow test. We used a two-tailed P value of 0.05 for the statistical analysis.

## Results

### Study population characteristics

**Figure 1:**
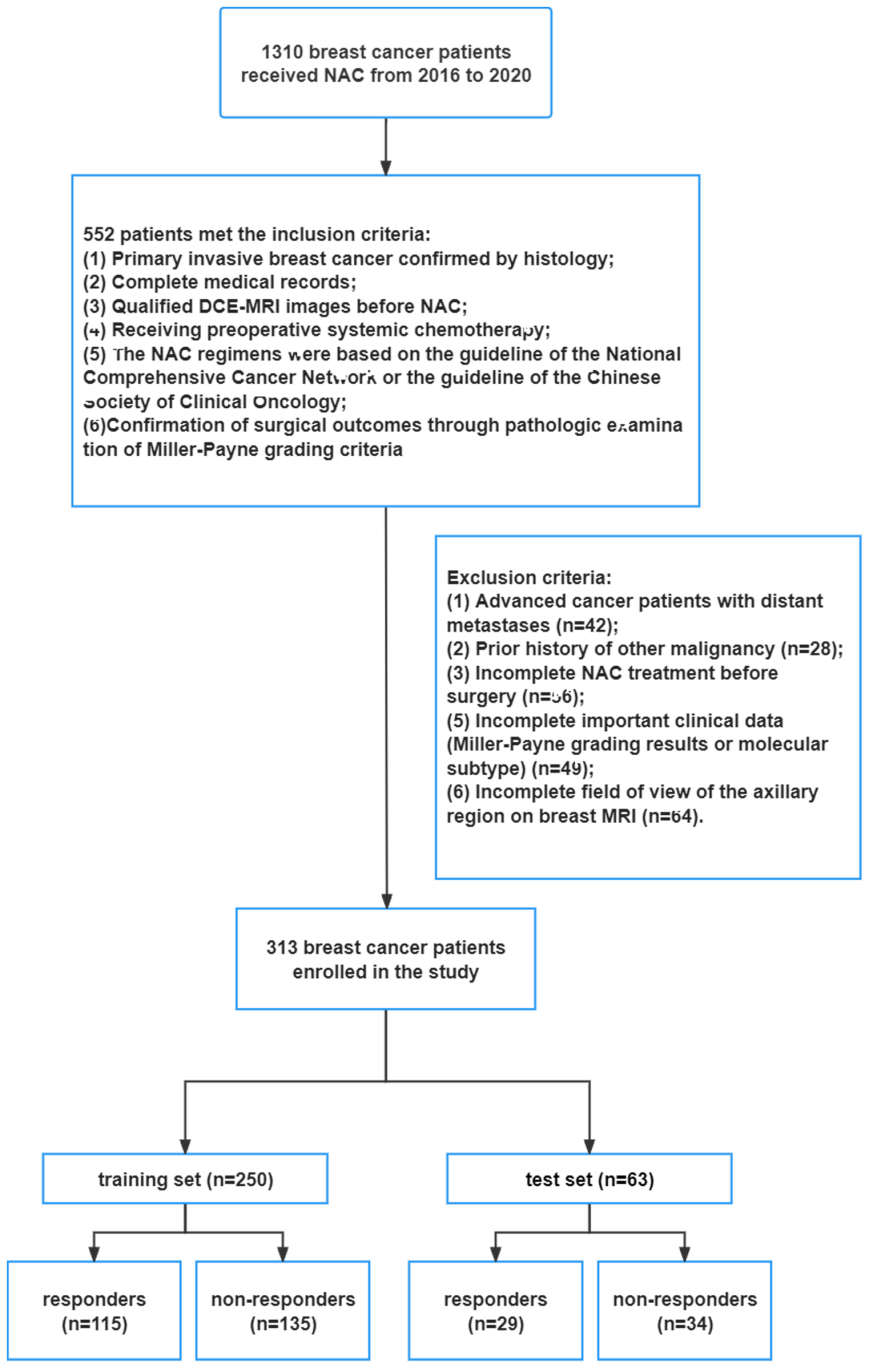
Flow chart of patient enrollment.

This study enrolled 313 patients with breast cancer from 2016 to 2021. The flowchart of the screening process is summarized in **Figure 1**. We randomly divided 313 breast patients into train and test sets at an 8:2 ratio. Based on pathological analysis of the surgical specimens, the Miller-Payne grading results were as follows: 16, 67, 86, 54, and 90 patients achieved G1, G2, G3, G4, and G5, respectively. 144 patients were classified as responders, and 169 patients were classified as non-responders.

**Table 1** lists the clinical characteristics of all patients. The ER status, PR status, Her-2 status, Ki-67 index, and clinical T stage showed a significant association with pathologic response after NAC in breast cancer patients. There was no significant difference between responders and non-responders in terms of age, menopausal status, and clinical N stage. Meanwhile, no statistically significant disparities were found in clinical parameters, including age, menopausal status, ER status, PR status, Her-2 status, Ki-67 index, and clinical T/N stages, between the train and test cohorts (shown in **Supplementary Table S2**).

**Table 1:** The clinical characteristics between Non-responders and responders.

Characteristics	Non-responders	Responders	P value
	169	144	
age, median (IQR)	51 (43, 58)	49.5 (42, 57)	0.206
Menopausal status, n (%)			0.248
Non-menopausal	84 (26.8%)	81 (25.9%)	
Post-menopausal	85 (27.2%)	63 (20.1%)	
ER, n (%)			< 0.001
Negative	39 (12.5%)	79 (25.2%)	
Positive	130 (41.5%)	65 (20.8%)	
PR, n (%)			< 0.001
Negative	55 (17.6%)	93 (29.7%)	
Positive	114 (36.4%)	51 (16.3%)	
HER-2, n (%)			< 0.001
Negative	130 (41.5%)	55 (17.6%)	
Positive	39 (12.5%)	89 (28.4%)	
Ki-67, median (IQR)	30 (20, 50)	50 (30, 60)	< 0.001
cT, n (%)			0.018
cT1	5 (1.6%)	16 (5.1%)	
cT2	85 (27.2%)	66 (21.1%)	
cT3	66 (21.1%)	46 (14.7%)	
cT4	13 (4.2%)	16 (5.1%)	
cN, n (%)			0.905
cN0	2 (0.6%)	1 (0.3%)	
cN1	148 (47.3%)	127 (40.6%)	
cN2	19 (6.1%)	16 (5.1%)	

### Deep learning features extraction and selection

**Figure 2:**
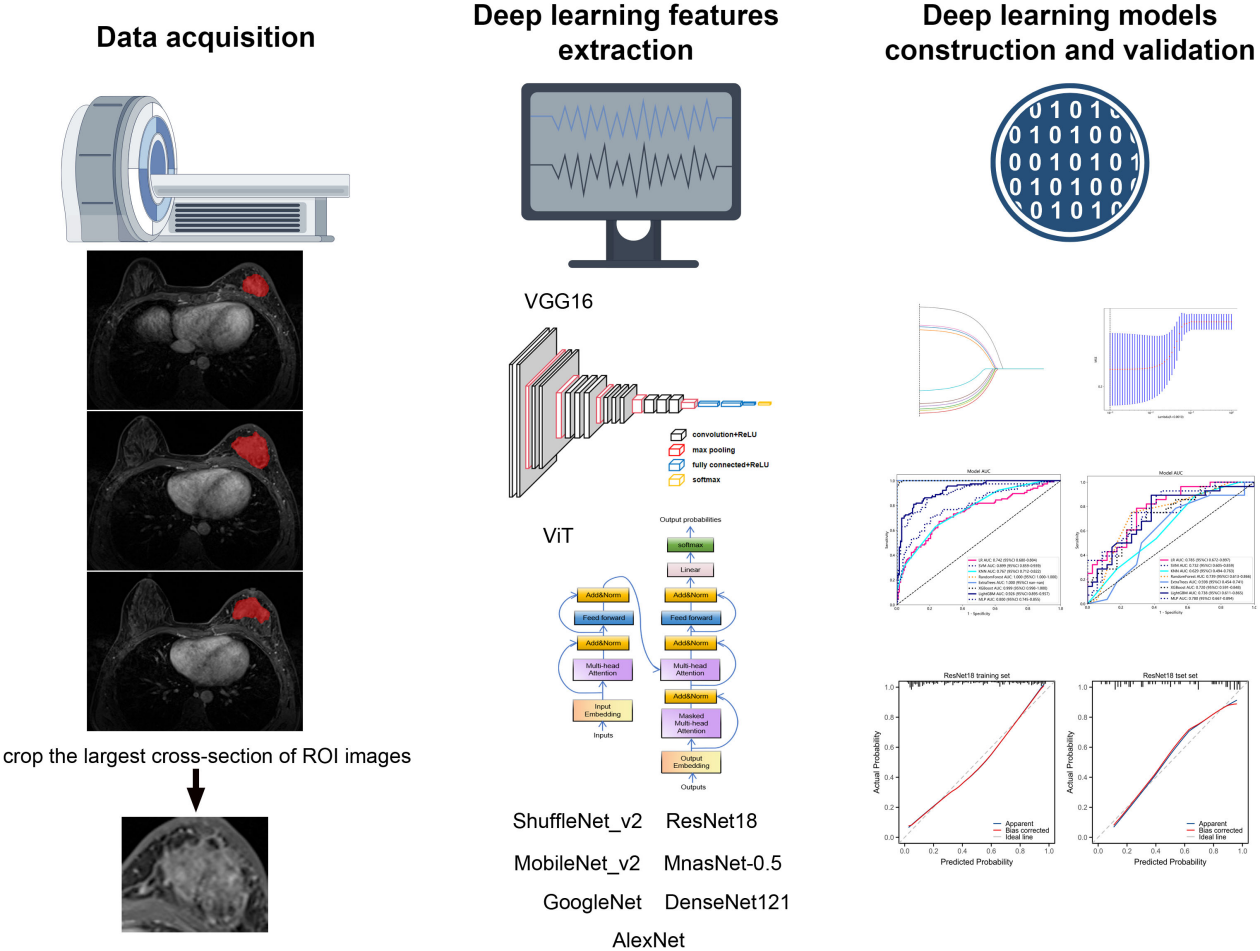
The flowchart of building deep learning radiomic models.

The flowchart of building the DLR signatures is summarized in **Figure 2**. Multiple deep learning frameworks, including ViT, VGG16, ShuffleNet_v2, ResNet18, MobileNet_v2, MnasNet-0.5, GoogleNet, DenseNet121, and AlexNet, were used for transfer learning of the classification model. The deep learning features were obtained from the final fully connected layer of the deep learning models, with 256 features extracted based on DCE-MRI data for each patient of each deep learning model. The Pearson correlation coefficient analysis and subsequent LASSO regression analysis were conducted to eliminate redundant and irrelevant features. As an example, 10 features and 11 features were chosen to construct classification model in ViT model and VGG16 model, respectively. The screened features were used for subsequent construction of classification model. The detailed selection features of deep learning models were shown in **Supplementary Table S3**.

### Deep learning models construction and validation

We analyzed the performance of SVM, KNN, RandomForest, ExtraTress, XGBoost, LightGBM, and MLP to construct classification models for predicting pathologic response following NAC in breast cancer patients. The detailed results of the models are shown in **Table 2**.

**Table 2:** The detailed results of different classifiers among various deep models for predicting pathologic response following NAC in breast cancer patients.

Deep learning model	Model		AUC	95% CI	Sensitivity	Specificity	PPV	NPV
ViT	SVM	train	0.90	0.86-0.94	0.69	0.92	0.88	0.78
		test	0.73	0.61-0.86	0.54	0.77	0.65	0.67
	KNN	train	0.77	0.71-0.82	0.63	0.76	0.69	0.71
		test	0.63	0.49-0.76	0.54	0.59	0.52	0.61
	RandomForest	train	1.00	1.00-1.00	0.95	1.00	1.00	0.96
		test	0.74	0.61-0.87	0.50	0.82	0.70	0.66
	ExtraTrees	train	1.00	1.00-1.00	1.00	1.00	1.00	1.00
		test	0.59	0.45-0.74	0.32	0.71	0.47	0.56
	XGBoost	train	0.99	1.00-1.00	0.98	0.985	0.983	0.985
		test	0.72	0.59-0.85	0.61	0.67	0.61	0.67
	LightGBM	train	0.93	0.89-0.96	0.80	0.89	0.86	0.84
		test	0.74	0.61-0.87	0.57	0.73	0.64	0.676
	MLP	train	0.80	0.74-0.85	0.59	0.86	0.78	0.71
		test	0.78	0.67-0.89	0.50	0.79	0.67	0.66
VGG16	SVM	train	0.92	0.88-0.95	0.76	0.89	0.85	0.81
		test	0.76	0.63-0.90	0.82	0.77	0.74	0.84
	KNN	train	0.82	0.77-0.86	0.65	0.85	0.78	0.74
		test	0.70	0.57-0.83	0.68	0.71	0.66	0.73
	RandomForest	train	1.00	1.00-1.00	0.96	1.00	1.00	0.97
		test	0.67	0.54-0.81	0.50	0.71	0.58	0.63
	ExtraTrees	train	1.00	1.00-1.00	1.00	1.00	1.00	1.00
		test	0.64	0.50-0.78	0.57	0.74	0.64	0.68
	XGBoost	train	1.00	1.00-1.00	0.99	1.00	1.00	0.99
		test	0.71	0.57-0.84	0.57	0.74	0.64	0.68
	LightGBM	train	0.97	0.95-0.99	0.85	0.95	0.93	0.88
		test	0.67	0.54-0.81	0.57	0.62	0.55	0.64
	MLP	train	0.85	0.80-0.90	0.66	0.82	0.76	0.74
		test	0.79	0.67-0.90	0.75	0.79	0.75	0.79
ShuffleNet_v2	SVM	train	0.92	0.89-0.96	0.79	0.92	0.89	0.84
		test	0.81	0.70-0.92	0.55	0.91	0.84	0.71
	KNN	train	0.80	0.74-0.85	0.75	0.73	0.70	0.77
		test	0.70	0.57-0.83	0.62	0.71	0.64	0.69
	RandomForest	train	1.00	1.00-1.00	0.94	1.00	1.00	0.95
		test	0.65	0.51-0.79	0.45	0.82	0.68	0.64
	ExtraTrees	train	1.00	1.00-1.00	1.00	1.00	1.00	1.00
		test	0.67	0.54-0.81	0.45	0.79	0.65	0.63
	XGBoost	train	1.00	1.00-1.00	1.00	1.00	1.00	1.00
		test	0.77	0.65-0.89	0.55	0.85	0.76	0.69
	LightGBM	train	0.94	0.92-0.97	0.80	0.94	0.92	0.85
		test	0.74	0.61-0.86	0.41	0.82	0.67	0.62
	MLP	train	0.84	0.80-0.89	0.67	0.90	0.85	0.76
		test	0.81	0.69-0.92	0.45	0.97	0.93	0.67
ResNet18	SVM	train	0.96	0.94-0.98	0.88	0.94	0.93	0.90
		test	0.81	0.70-0.92	0.72	0.82	0.78	0.78
	KNN	train	0.86	0.82-0.90	0.62	0.91	0.86	0.74
		test	0.64	0.51-0.78	0.52	0.68	0.58	0.62
	RandomForest	train	1.00	0.99-1.00	0.97	0.99	0.98	0.98
		test	0.61	0.48-0.75	0.45	0.65	0.52	0.58
	ExtraTrees	train	1.00	1.00-1.00	1.00	1.00	1.00	1.00
		test	0.61	0.47-0.75	0.45	0.79	0.65	0.63
	XGBoost	train	1.00	1.00-1.00	1.00	1.00	1.00	1.00
		test	0.59	0.44-0.73	0.69	0.47	0.53	0.64
	LightGBM	train	0.96	0.94-0.98	0.80	0.96	0.94	0.85
		test	0.59	0.44-0.73	0.31	0.59	0.39	0.50
	MLP	train	0.87	0.82-0.91	0.77	0.81	0.77	0.80
		test	0.87	0.78-0.96	0.83	0.74	0.73	0.83
MobileNet_v2	SVM	train	0.91	0.87-0.95	0.76	0.90	0.87	0.82
		test	0.72	0.59-0.85	0.52	0.91	0.83	0.69
	KNN	train	0.78	0.72-0.83	0.69	0.75	0.70	0.74
		test	0.59	0.46-0.73	0.35	0.65	0.46	0.54
	RandomForest	train	1.00	1.00-1.00	0.97	0.99	0.99	0.98
		test	0.63	0.49-0.76	0.35	0.77	0.56	0.58
	ExtraTrees	train	1.00	1.00-1.00	1.00	1.00	1.00	1.00
		test	0.57	0.42-0.71	0.41	0.65	0.50	0.56
	XGBoost	train	1.00	1.00-1.00	1.00	1.00	1.00	1.00
		test	0.70	0.57-0.83	0.48	0.74	0.61	0.63
	LightGBM	train	0.94	0.91-0.97	0.83	0.94	0.92	0.87
		test	0.70	0.57-0.83	0.45	0.88	0.77	0.65
	MLP	train	0.83	0.78-0.88	0.63	0.85	0.78	0.73
		test	0.74	0.62-0.87	0.62	0.79	0.72	0.71
MnasNet-0.5	SVM	train	0.87	0.83-0.92	0.61	0.92	0.86	0.74
		test	0.65	0.52-0.79	0.38	0.74	0.55	0.58
	KNN	train	0.77	0.71-0.83	0.66	0.76	0.70	0.73
		test	0.55	0.41-0.69	0.41	0.68	0.52	0.58
	RandomForest	train	1.00	1.00-1.00	0.97	0.99	0.99	0.97
		test	0.58	0.44-0.72	0.41	0.65	0.50	0.56
	ExtraTrees	train	1.00	1.00-1.00	1.00	1.00	1.00	1.00
		test	0.68	0.55-0.81	0.45	0.74	0.59	0.61
	XGBoost	train	1.00	1.00-1.00	1.00	0.99	0.99	1.00
		test	0.66	0.51-0.80	0.55	0.79	0.70	0.68
	LightGBM	train	0.92	0.89-0.96	0.75	0.91	0.88	0.81
		test	0.75	0.63-0.88	0.41	0.88	0.75	0.64
	MLP	train	0.79	0.73-0.84	0.54	0.85	0.76	0.69
		test	0.65	0.50-0.79	0.35	0.65	0.46	0.54
GoogleNet	SVM	train	0.93	0.90-0.96	0.74	0.91	0.88	0.81
		test	0.80	0.68-0.91	0.62	0.77	0.69	0.70
	KNN	train	0.80	0.74-0.85	0.69	0.80	0.75	0.75
		test	0.66	0.53-0.80	0.59	0.77	0.68	0.68
	RandomForest	train	1.00	0.99-1.00	0.96	0.99	0.98	0.96
		test	0.69	0.56-0.82	0.52	0.74	0.63	0.64
	ExtraTrees	train	1.00	1.00-1.00	1.00	1.00	1.00	1.00
		test	0.65	0.51-0.78	0.55	0.65	0.57	0.63
	XGBoost	train	1.00	1.00-1.00	0.99	1.00	1.00	0.99
		test	0.68	0.55-0.81	0.59	0.71	0.63	0.67
	LightGBM	train	0.95	0.92-0.97	0.79	0.93	0.91	0.84
		test	0.74	0.61-0.86	0.69	0.68	0.65	0.72
	MLP	train	0.84	0.79-0.88	0.64	0.84	0.77	0.74
		test	0.79	0.67-0.90	0.62	0.77	0.69	0.70
DenseNet121	SVM	train	0.96	0.94-0.98	0.70	0.80	0.75	0.76
		test	0.75	0.63-0.87	0.62	0.77	0.69	0.70
	KNN	train	0.82	0.77-0.87	0.97	0.99	0.99	0.97
		test	0.72	0.60-0.85	0.38	0.79	0.61	0.60
	RandomForest	train	1.00	1.00-1.00	1.00	1.00	1.00	1.00
		test	0.67	0.54-0.80	0.48	0.79	0.67	0.64
	ExtraTrees	train	1.00	1.00-1.00	1.00	1.00	1.00	1.00
		test	0.69	0.56-0.82	0.69	0.68	0.65	0.72
	XGBoost	train	1.00	1.00-1.00	0.79	0.93	0.90	0.84
		test	0.73	0.60-0.86	0.52	0.71	0.60	0.63
	LightGBM	train	0.95	0.92-0.97	0.70	0.85	0.79	0.77
		test	0.72	0.59-0.84	0.62	0.74	0.67	0.69
	MLP	train	0.88	0.83-0.92	0.70	0.80	0.75	0.76
		test	0.74	0.62-0.87	0.62	0.77	0.69	0.70
AlexNet	SVM	train	0.94	0.92-0.97	0.79	0.93	0.91	0.84
		test	0.84	0.74-0.94	0.69	0.82	0.77	0.76
	KNN	train	0.83	0.78-0.88	0.76	0.79	0.75	0.79
		test	0.62	0.47-0.76	0.62	0.59	0.56	0.65
	RandomForest	train	1.00	1.00-1.00	0.97	1.00	1.00	0.97
		test	0.70	0.57-0.83	0.52	0.74	0.63	0.64
	ExtraTrees	train	1.00	1.00-1.00	1.00	1.00	1.00	1.00
		test	0.59	0.45-0.73	0.31	0.71	0.47	0.55
	XGBoost	train	1.00	1.00-1.00	0.99	1.00	1.00	0.99
		test	0.74	0.61-0.87	0.72	0.74	0.70	0.76
	LightGBM	train	0.95	0.93-0.98	0.78	0.95	0.93	0.84
		test	0.62	0.48-0.77	0.59	0.71	0.63	0.67
	MLP	train	0.87	0.82-0.91	0.73	0.88	0.84	0.80
		test	0.84	0.73-0.94	0.72	0.88	0.84	0.79

Taking the ViT deep learning model as an example, in the train cohort, the AUC for SVM, KNN, RandomForest, ExtraTress, XGBoost, LightGBM, and MLP were recorded at 0.90, 0.77, 1.00, 1.00, 0.99, 0.93, and 0.80, respectively. Within the test cohort, these values were observed as 0.73 for SVM, 0.63 for KNN, 0.74 for RandomForest, 0.59 for ExtraTrees, 0.72 for XGBoost, 0.74 for LightGBM, and 0.78 for MLP, respectively. The MLP classification model exhibited good performance with an AUC of 0.80 (95% CI, 0.74 - 0.85) and 0.78 (95% CI, 0.67 - 0.89) in train and test groups, respectively. The Delong’s test was utilized to access the disparities in predictive performance between MLP and other alternative models in the test cohort. The predictive capacity of MLP model is better than that of the KNN (*p* < 0.01) and ExtraTrees (*p* =0.02) models; however, it exhibits no statistically significant differences when compared with other models.

**Figure 3:**
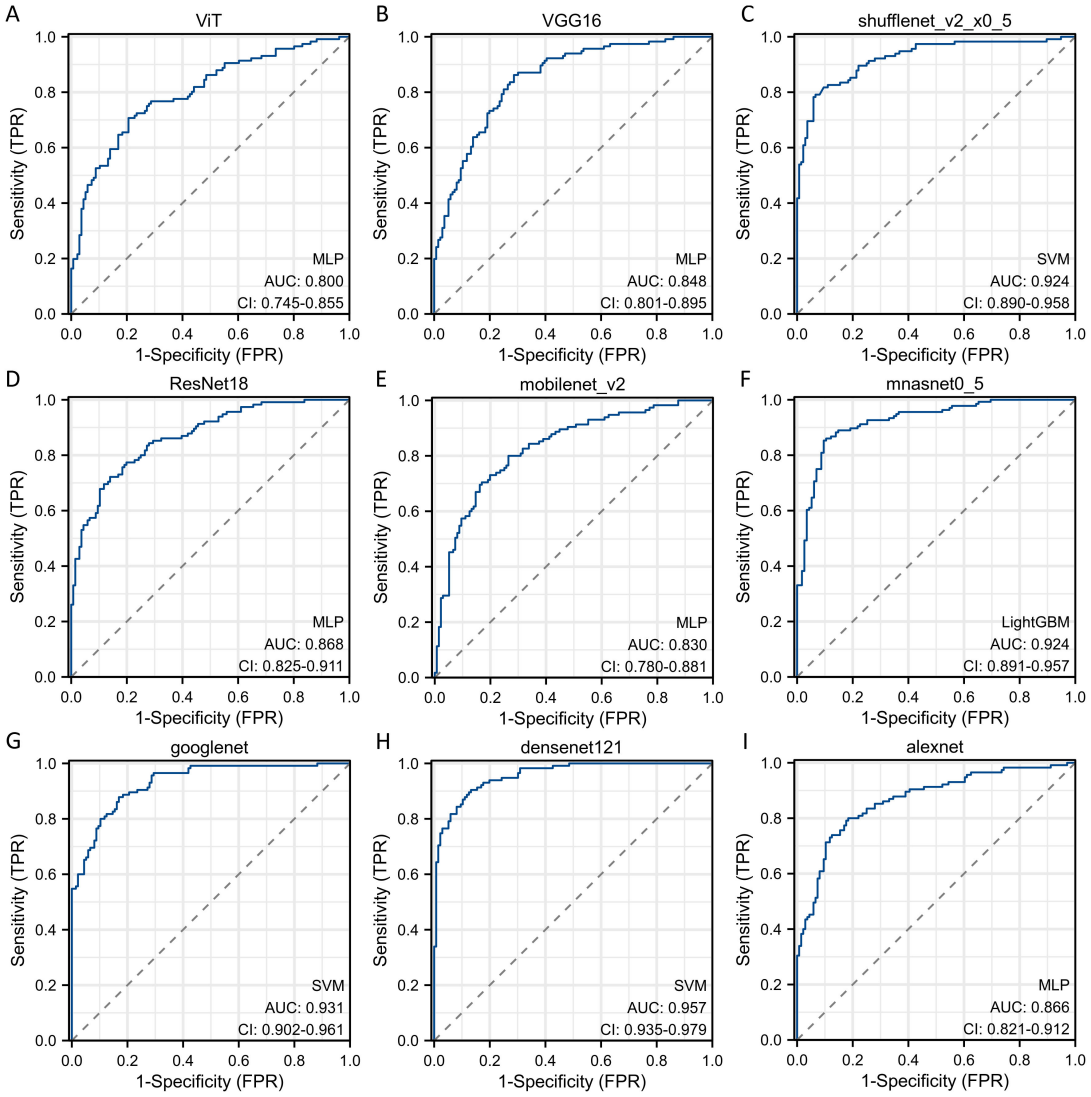
The ROC curves of different deep learning models for predicting pathological response of breast cancer patients after NAC in train cohort. **(A)** ROC curve for ViT model; **(B)** ROC curve for VGG16 model; **(C)** ROC curve for ShuffleNet_v2 model; **(D)** ROC curve for ResNet18 model; **(E)** ROC curve for MobileNet_v2 model; **(F)** ROC curve for MnasNet-0.5 model; **(G)** ROC curve for GoogleNet model; **(H)** ROC curve for DenseNet121 model; **(I)** ROC curve for AlexNet model.

**Figure 4:**
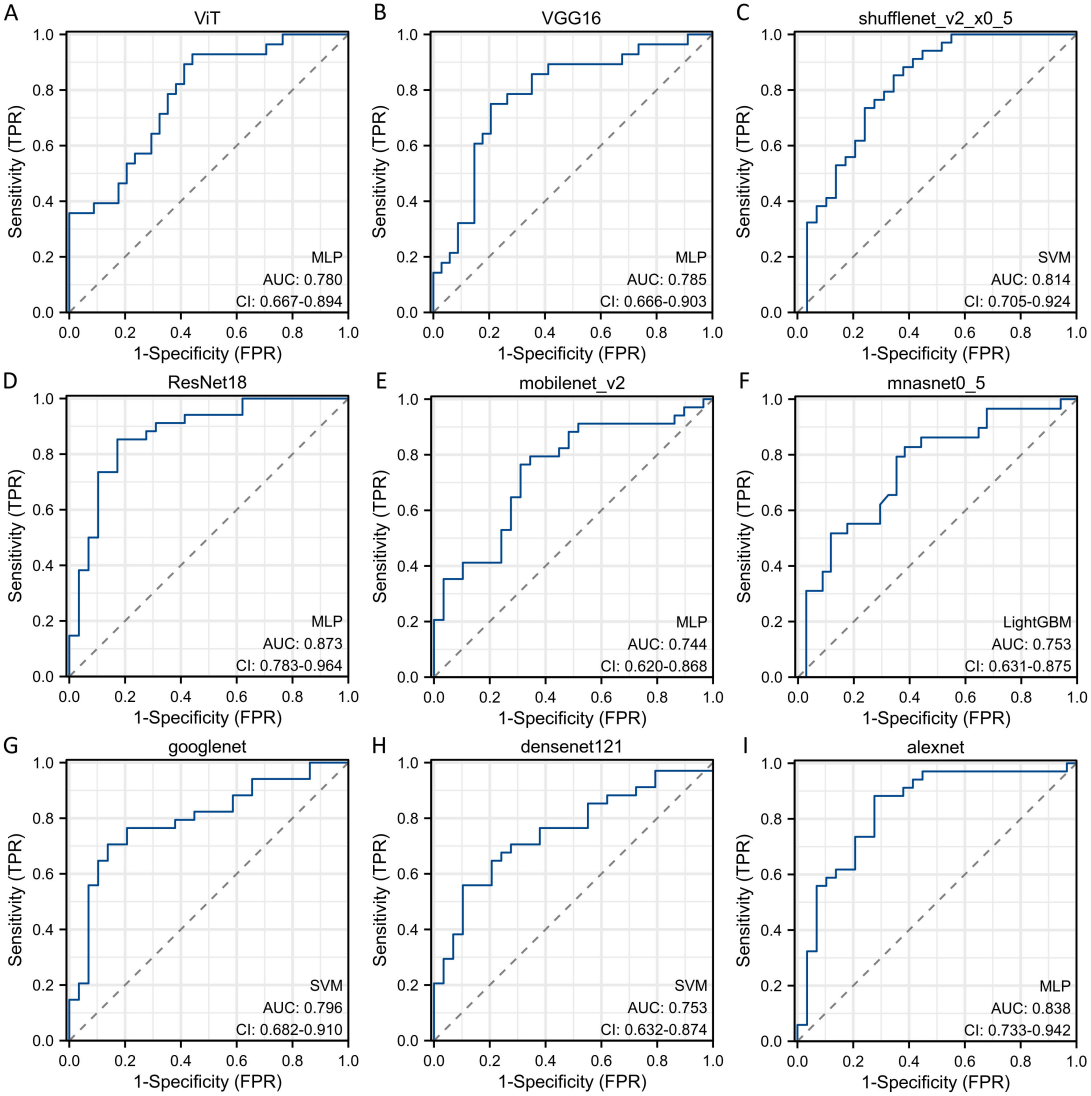
The ROC curves of different deep learning models for predicting pathological response of breast cancer patients after NAC in test cohort. **(A)** ROC curve for ViT model; **(B)** ROC curve for VGG16 model; **(C)** ROC curve for ShuffleNet_v2 model; **(D)** ROC curve for ResNet18 model; **(E)** ROC curve for MobileNet_v2 model; **(F)** ROC curve for MnasNet-0.5 model; **(G)** ROC curve for GoogleNet model; **(H)** ROC curve for DenseNet121 model; **(I)** ROC curve for AlexNet model.

**Figure 5:**
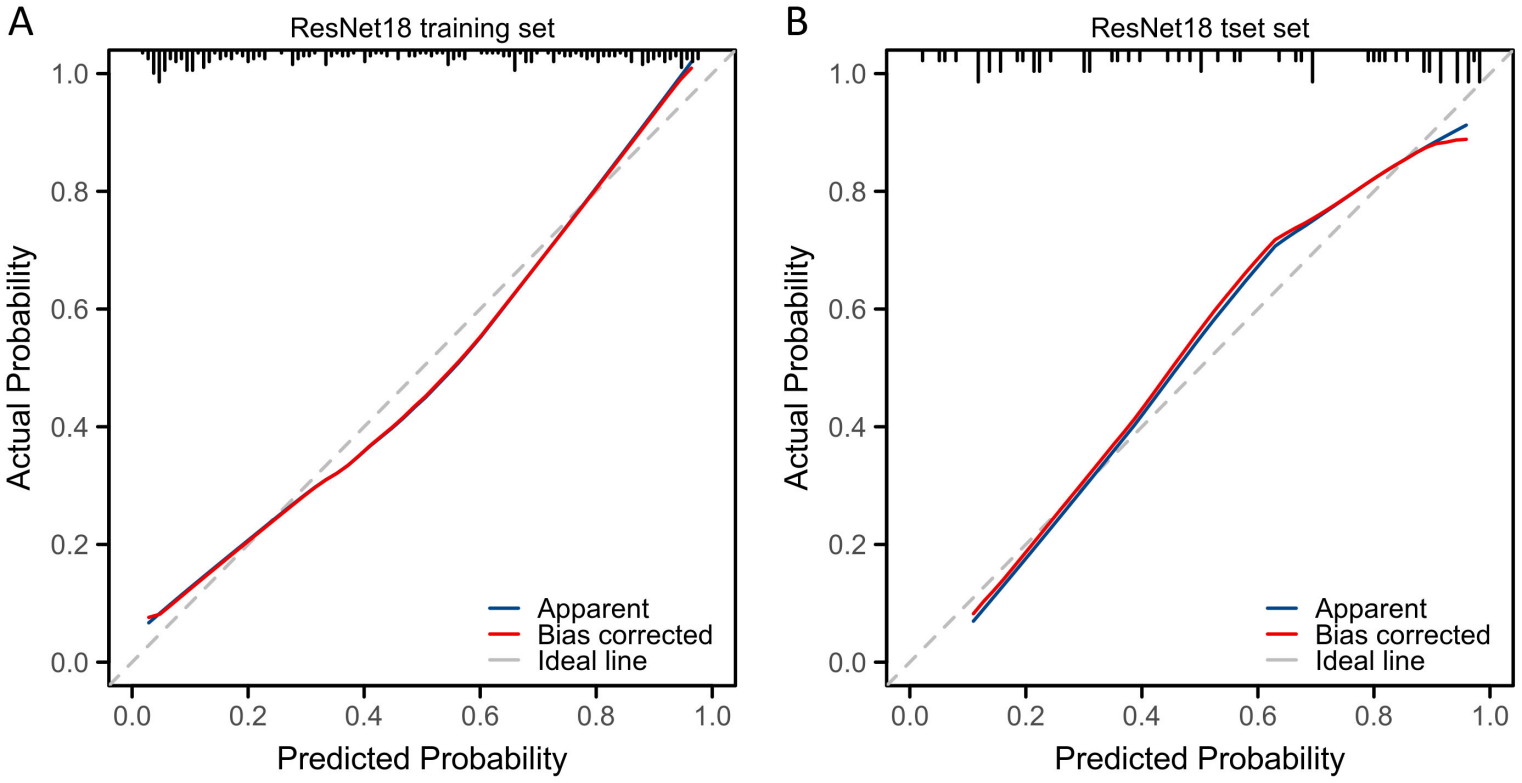
The calibration curves of radiomic signature based on different classification models. **(A)** Calibration curve of ResNet18 model in the train set; **(B)** Calibration curve of ResNet18 model in the test set.

Sequentially, we utilized various deep learning models to extract features and subsequently constructed machine learning models. Based on the performance of different machine learning models, we selected the classifiers with the best performance. The specific results of the best-performing classifiers among various deep models are presented in **Table 3**. The comparative performance of diverse deep learning models exhibits substantial equivalence, although ResNet18 and AlexNet demonstrates marginally superior outcomes. The ROC curves of different deep learning models are shown in detail in **Figures 3** and **4**. In the training set, the sensitivity, specificity, PPV, and NPV of the ResNet18 model are 0.77, 0.81, 0.77, and 0.80, respectively. In the test set, the sensitivity, specificity, PPV, and NPV of the ResNet18 model are 0.83, 0.74, 0.73, and 0.83, respectively. In the internal validation set, the DeLong test revealed that the predictive performance of ResNet18 was significantly superior to MobileNet_v2 (p= 0.04), MnasNet-0.5 (p=0.04), and DenseNet121 (p=0.04), with statistical significance. The calibration curves for ResNet18 consistently showed agreement both in the train set (illustrated in **Figure 5A**) and the test set (illustrated in **Figure 5B**).

**Table 3:** The best-performing classifiers among various deep models.

Model	Best-performing classifier		AUC	95% CI	Sensitivity	Specificity	PPV	NPV
ViT	MLP	train	0.80	0.74-0.85	0.59	0.86	0.78	0.71
		test	0.78	0.67-0.89	0.50	0.79	0.67	0.66
VGG16	MLP	train	0.85	0.80-0.90	0.66	0.82	0.76	0.74
		test	0.79	0.67-0.90	0.75	0.79	0.75	0.79
ShuffleNet_v2	SVM	train	0.92	0.89-0.95	0.79	0.92	0.89	0.84
		test	0.81	0.70-0.92	0.55	0.92	0.84	0.71
ResNet18	MLP	train	0.87	0.82-0.91	0.77	0.81	0.77	0.80
		test	0.87	0.78-0.96	0.83	0.74	0.73	0.83
MobileNet_v2	MLP	train	0.83	0.78-0.88	0.63	0.85	0.78	0.73
		test	0.74	0.62-0.87	0.62	0.79	0.72	0.71
MnasNet-0.5	LightGBM	train	0.92	0.89-0.96	0.75	0.91	0.88	0.81
		test	0.75	0.63-0.88	0.41	0.88	0.75	0.64
GoogleNet	SVM	train	0.93	0.90-0.96	0.74	0.91	0.88	0.81
		test	0.80	0.68-0.91	0.62	0.77	0.69	0.70
DenseNet121	SVM	train	0.96	0.94-0.98	0.70	0.80	0.75	0.76
		test	0.75	0.63-0.87	0.62	0.77	0.69	0.70
AlexNet	MLP	train	0.87	0.82-0.91	0.73	0.88	0.84	0.80
		test	0.84	0.73-0.94	0.72	0.88	0.84	0.79

## Discussion

NAC stands as the established therapeutic approach for both locally advanced and early invasive breast cancer patients who aimed at facilitating breast-conserving surgeries. The identification of a reliable method for predicting sensitivity to NAC before surgical intervention holds significant importance in treatment planning. This assessment profoundly influences the choice between initiating NAC followed by surgery or proceeding directly to surgery without prior NAC administration. Radiomics has emerged as a burgeoning domain within cancer treatment. Typically, quantitative data extracted from images is automatically analyzed to correlate with malignant biological properties, therapeutic efficacy, and clinical prognosis. This approach offers a promising avenue for delivering tailored precision therapy in a non-invasive manner. With advancements in deep learning radiomics and associated data processing tools, the interpretation and utilization of data in clinical contexts have become more accessible.

In this study, we enrolled 313 breast cancer patients who had complete DCE-MRI data and underwent NAC followed by breast surgery. Various deep learning frameworks, such as ViT, VGG16, ShuffleNet_v2, ResNet18, MobileNet_v2, MnasNet-0.5, GoogleNet, DenseNet121, and AlexNet, were utilized for transfer learning to develop the classification model. Deep learning features were extracted from the fully connected layer and used to construct classification models. ResNet18 exhibited superior performance, with an AUC of 0.87 (95% CI: 0.82 - 0.91) and 0.87 (95% CI: 0.78 - 0.96) in the train and test cohorts, respectively.

In the realm of medical image analysis, deep learning-driven radiomic features have demonstrated superior performance. Li (11) et al. recruited 95 breast cancer patients to construct a DLR model that integrates pre-treatment and early-treatment DCE-MRI data for predicting pCR to NAC. The AUC of DLR was 0.64 for pre-treatment, 0.88 for early-treatment, and 0.90 for combined data. In a multicenter retrospective study, 1262 patients were included in order to develop a novel tool for predicting pCR of breast cancer to NAC (12). The stacking model, which integrates pre-, post-, and delta-models based on traditional radiomic features and DLR features, achieved AUC values of 0.89, 0.92, and 0.89 in the external validation cohorts, respectively. Traditional methods are simple, conventional, and not black-box models. However, models based solely on traditional radiomic features do not show ideal predictive performance, while models based on multi-omics, multi-temporal data, traditional radiomic features, and deep learning features demonstrate better predictive power, albeit with a more complex process.

We conducted experiments to explore the base DLR model’s performance without machine learning step. The results are included in the **Supplementary Table S4**, where we provide a detailed analysis of the performance metrics for each approach. The findings indicate that while the base DLR models showed suboptimal performance, we further conducted a two-stage system with deep learning (for feature generation), and machine learning (for feature transformation followed by classifiers). The integration of machine learning with feature transformation significantly enhances the overall performance, justifying the need for the proposed approach.

Our study has the following advantages. At first, it focused on identifying patients with Miller-Payne grade 4-5 who responded better to NAC, rather than solely predicting pCR status. This approach allowed us to select patients who may benefit from NAC. Additionally, it enabled patients who were insensitive to NAC to proceed directly to surgical therapy, avoiding excessive therapy and potentially losing the opportunity for surgery due to disease progression after NAC. Simultaneously, pCR indicated complete pathological remission of both the metastatic axillary lymph nodes and the primary breast tumor. Predicting pCR through radiomics requires delineation and feature extraction of ROI separately for the metastatic lymph nodes and the primary breast tumor, which undoubtedly increased the complexity of the radiomics model, affecting its reproducibility and practical application. Finally, previous studies have mostly focused on building models for predicting NAC response in breast cancer patients using MRI parameters and traditional radiomics. In contrast, we explored multiple deep learning models for predicting NAC response and found ResNet18 demonstrated excellent performance, achieving an AUC of 0.87 and 0.87 in the train and test cohorts, respectively. Despite the strengths of our study, there is a lot of room for enhancement. Initially, a singular imaging protocol is utilized for pre-treatment NAC. Although DCE-MRI of the breast stood out as the most distinctive, the multiparametric MRI, including T2WI and DWI, might provide more comprehensive and unbiased data. Furthermore, this study was limited by its retrospective and single-center nature. A prospective, multicenter investigation could help in creating a universal prognostic model applicable to various clinical scenarios.

Using pre-treatment DCE-MRI images, our study trained multiple deep models and developed the best-performing DLR model for predicting pathologic response of NAC in breast cancer patients. The model demonstrated excellent performance in both the train and test cohorts. As a result, this prognostic tool provides a dependable and impartial basis for effectively identifying breast cancer patients who are most likely to benefit from NAC before its initiation. At the same time, it can also identify those patients who are insensitive to NAC, allowing them to proceed directly to surgical treatment and prevent the risk of losing the opportunity for surgery due to disease progression after NAC.

## Data Availability

The raw data supporting the conclusions of this article will be made available by the authors, without undue reservation.

## Supplementary material

Supplementary Table 3 The detailed selection features of deep learning models.
