# Supplementary material for: Deep learning model for the early prediction of pathologic response following neoadjuvant chemotherapy in breast cancer patients using dynamic contrast-enhanced MRI
Source: Front Oncol. 2025 Feb 25;15:1491843. doi: 10.3389/fonc.2025.1491843 (PMC11893424; doi:10.3389/fonc.2025.1491843)
Supplement: Supplementary file 1 [file Table1.docx]

Supplementary Table 1. The deep learning model parameters.

| Model | Deep learning model parameters |
| --- | --- |
| Vision Transformer (ViT) | Input Image Size: 224x224  Patch Size: 16x16  Embedding Dimension: 768  Number of Layers (Transformer Blocks): 12  Number of Attention Heads: 12  Feedforward Size: 3072  Learning Rate: 0.001  Batch Size: 32  Optimizer: AdamW with weight decay |
| VGG16 | Input Image Size: 224x224  Number of Convolutional Layers: 13  Fully Connected Layers: 3  Learning Rate: 0.001  Batch Size: 32  Optimizer: SGD with momentum  Dropout Rate: 0.5 |
| ShuffleNetV2 | Input Image Size: 224x224  Learning Rate: 0.001  Batch Size: 32  Optimizer: SGD with momentum  Momentum: 0.9 |
| ResNet18 | Input Image Size: 224x224  Learning Rate: 0.001  Batch Size: 32  Optimizer: SGD with momentum  Initial Learning Rate: 0.1, with a step decay (e.g., divide by 10 every 30 epochs) |
| MobileNetV2 | Input Image Size: 224x224  Learning Rate: 0.001  Batch Size: 32  Optimizer: Adam  Dropout Rate: 0.2 |
| MnasNet0.5 | Input Image Size: 224x224  Learning Rate: 0.001  Batch Size: 32  Optimizer: Adam  Momentum: 0.9  Dropout Rate: 0.3 |
| GoogleNet | Input Image Size: 224x224  Learning Rate: 0.001  Batch Size: 32  Optimizer: SGD with momentum  Momentum: 0.9  Learning Rate Decay: Exponential decay |
| DenseNet121 | Input Image Size: 224x224  Learning Rate: 0.001  Batch Size: 32  Optimizer: SGD with momentum  Momentum: 0.9  Dropout Rate: 0.2 |
| AlexNet | Input Image Size: 224x224  Learning Rate: 0.001  Batch Size: 32  Optimizer: SGD with momentum  Momentum: 0.9  Dropout Rate: 0.5 (in fully connected layers) |
