# Supplementary material for: Deep learning model for the early prediction of pathologic response following neoadjuvant chemotherapy in breast cancer patients using dynamic contrast-enhanced MRI
Source: Front Oncol. 2025 Feb 25;15:1491843. doi: 10.3389/fonc.2025.1491843 (PMC11893424; doi:10.3389/fonc.2025.1491843)
Supplement: Supplementary file 2 [file Table2.docx]

| Characteristics | Train cohort | Test cohort | P value |
| --- | --- | --- | --- |
|  | 250 | 63 |  |
| age, median (IQR) | 50 (43, 58) | 49 (43.5, 56) | 0.579 |
| Menopausal status, n (%) |  |  | 0.953 |
| Non-menopausal | 132 (42.2%) | 33 (10.5%) |  |
| Post-menopausal | 118 (37.7%) | 30 (9.6%) |  |
| ER, n (%) |  |  | 0.513 |
| Negative | 92 (29.4%) | 26 (8.3%) |  |
| Postive | 158 (50.5%) | 37 (11.8%) |  |
| PR, n (%) |  |  | 0.953 |
| Negative | 118 (37.7%) | 30 (9.6%) |  |
| Postive | 132 (42.2%) | 33 (10.5%) |  |
| HER-2, n (%) |  |  | 0.827 |
| Negative | 147 (47%) | 38 (12.1%) |  |
| Postive | 103 (32.9%) | 25 (8%) |  |
| Ki-67, median (IQR) | 40 (20, 50) | 50 (30, 50) | 0.129 |
| cT, n (%) |  |  | 0.898 |
| cT1 | 17 (5.4%) | 4 (1.3%) |  |
| cT2 | 123 (39.3%) | 28 (8.9%) |  |
| cT3 | 87 (27.8%) | 25 (8%) |  |
| cT4 | 23 (7.3%) | 6 (1.9%) |  |
| cN, n (%) |  |  | 0.849 |
| cN0 | 2 (0.6%) | 1 (0.3%) |  |
| cN1 | 220 (70.3%) | 55 (17.6%) |  |
| cN2 | 28 (8.9%) | 7 (2.2%) |  |
| Pathologic response, n (%) |  |  | 0.996 |
| 0 | 135 (43.1%) | 34 (10.9%) |  |
| 1 | 115 (36.7%) | 29 (9.3%) |  |

Supplementary Table 2. The clinical characteristics between train and test cohort.
