# Supplementary material for: Deep learning model for the early prediction of pathologic response following neoadjuvant chemotherapy in breast cancer patients using dynamic contrast-enhanced MRI
Source: Front Oncol. 2025 Feb 25;15:1491843. doi: 10.3389/fonc.2025.1491843 (PMC11893424; doi:10.3389/fonc.2025.1491843)
Supplement: Supplementary file 4 [file Table4.docx]

Supplementary Table 4. The results of base DLR model's performance without machine learning step

|  |  | **AUC** | **Sensitivity** | **Specificity** | **PPV** | **NPV** |
| --- | --- | --- | --- | --- | --- | --- |
| ViT | train cohort | 0.54 | 0.35 | 0.75 | 0.55 | 0.56 |
|  | Test cohort | 0.60 | 0.78 | 0.46 | 0.68 | 0.6 |
| VGG16 | train cohort | 0.81 | 0.72 | 0.77 | 0.73 | 0.75 |
|  | Test cohort | 0.53 | 0.82 | 0.42 | 0.67 | 0.61 |
| ShuffleNet_v2 | train cohort | 0.61 | 0.49 | 0.69 | 0.59 | 0.60 |
|  | Test cohort | 0.47 | 0.42 | 0.65 | 0.64 | 0.44 |
| ResNet18 | train cohort | 0.60 | 0.57 | 0.62 | 0.57 | 0.62 |
|  | Test cohort | 0.56 | 0.97 | 0.31 | 0.67 | 0.89 |
| MobileNet_v2 | train cohort | 0.64 | 0.88 | 0.35 | 0.55 | 0.77 |
|  | Test cohort | 0.54 | 1.00 | 0.15 | 0.63 | 1.00 |
| MnasNet-0.5 | train cohort | 0.49 | 0.64 | 0.45 | 0.51 | 0.58 |
|  | Test cohort | 0.49 | 0.32 | 0.85 | 0.75 | 0.46 |
| GoogleNet | train cohort | 0.74 | 0.70 | 0.73 | 0.69 | 0.73 |
|  | Test cohort | 0.61 | 0.61 | 0.62 | 0.69 | 0.52 |
| DenseNet121 | train cohort | 0.60 | 0.50 | 0.69 | 0.60 | 0.61 |
|  | Test cohort | 0.62 | 0.89 | 0.34 | 0.67 | 0.69 |
| AlexNet | train cohort | 0.71 | 0.66 | 0.71 | 0.67 | 0.70 |
|  | Test cohort | 0.55 | 0.76 | 0.42 | 0.66 | 0.55 |
